# Onion *Fusarium* Basal Rot Disease Control by Arbuscular Mycorrhizal Fungi and *Trichoderma harzianum*

**DOI:** 10.3390/plants13030386

**Published:** 2024-01-28

**Authors:** Abdulaziz Yağmur, Semra Demir, Sirel Canpolat, Younes Rezaee Danesh, Beatrice Farda, Rihab Djebaili, Loretta Pace, Marika Pellegrini

**Affiliations:** 1 Directorate of Plant Protection Central Research Institute, Ministry of Agriculture and Forestry, 06172 Ankara, Turkey; abdulaziz.yagmur@tarimorman.gov.tr (A.Y.); sirel.canpolat@tarimorman.gov.tr (S.C.); 2 Department of Plant Protection, Faculty of Agriculture, Van Yuzuncu Yil University, 65090 Van, Turkey; y.rdanesh@yahoo.com; 3 Department of Plant Protection, Faculty of Agriculture, Urmia University, Urmia 5756151818, Iran; 4 Department of Life, Health and Environmental Sciences, University of L’Aquila, 67100 L’Aquila, Italy; beatrice.farda@graduate.univaq.it (B.F.); loretta.pace@univaq.it (L.P.); marika.pellegrini@univaq.it (M.P.)

**Keywords:** *Allium cepa*, biocontrol, fusariosis, AMF, agricultural microbiology, sustainable agriculture

## Abstract

Soilborne pathogens reduce 60% of the yield of onion crops. A common fungal pathogen causing wilt disease and severe losses is *Fusarium* basal rot (FBR). In this study, the combination of Arbuscular Mycorrhizal Fungi (AMF) with *Trichoderma harzianum* was investigated against FBR. Onion samples were collected from the Ankara–Polatlı region. Among the isolates, isolate S6 was identified as *F. oxysporum* f. sp. *cepae* (FOC) using morphological and molecular methods and pathogenicity tests. Different combinations of AMF (*Funneliformis mosseae* pure strain and the commercial AMF) and *T. harzianum* were inoculated on susceptible onion cultivars (Seç, Gence, and Şampiyon). The effects of the treatments on FOC biocontrol were studied under growth chamber conditions. The results showed that Şampiyon was the most resistant, while Gence was the most susceptible to basal rot disease. Different colonization rates (8.91–24%), spore densities (16.4–50.4 spore/10 g soil), and the extent to which a plant needs mycorrhizal conditions to grow to its maximum potential (i.e., mycorrhizal dependencies—18.3–51.9%) were recorded by treatment. Both single and combined applications of AMF and *Trichoderma* applications suppressed FOC. Suppressive effects were more pronounced when the *F. mosseae* pure strain was used alone (when *F. mosseae* was used, disease severity decreased from 90 to 68%, *p* < 0.05). The *F. mosseae* pure strain also showed the best plant growth promotion and phosphorus content release. The results indicate an interesting potential use of *F. mosseae* and the combination of AMF with *T. harzianum* in the management of FOC in onions.

## 1. Introduction

Onion (*Allium cepa* L., Alliaceae family) is a common crop that is grown worldwide under a wide range of climatic conditions. With millions of tons of onions produced each year, the total global production of this product is substantial [1]. The top countries producing onions change from year to year, but the largest producers include India, Pakistan, China, the United States, and Russia, meeting both internal and foreign demand. Several onion cultivars are cultivated worldwide (e.g., yellow, red, and white). According to FAOSTAT, the production of dry onions and shallots (excluding dehydrated) in Türkiye and Italy in 2021 was 2,500,000 and 419,690 tons, respectively (FAOSTAT, https://www.fao.org/faostat/en/#data/QCL, accessed on 22 September 2023). Onion cultivation is a key aspect of global agriculture, providing an essential source of food and economic support for many communities. The crop’s adaptability and versatility in different culinary traditions contribute to its worldwide distribution [2]. Successful onion production requires effective weed and pest control, proper soil preparation, and adequate irrigation. Crop rotation is also essential to reduce the danger of soil-borne diseases [3].

Among soil-borne diseases, *Fusarium* basal rot (FBR) is an important concern for onion growers [4]. FBR is caused by various species of the genus *Fusarium*. Among the latter, *F. oxysporum* f. sp. *cepae* (FOC) is the major causal agent of root rot in onion production areas in many countries [5,6,7,8]. This disease primarily affects the bulbs of the plants and can result in significant economic losses for onion growers. Infection can start at the seedling stage, affects the whole life of a plant, and may continue during the storage period. Its effects on seed germination result in seedling death before and after emergence, as well as a decrease in head weight [9]. In severe infections, the outer tissues are also infected and covered by the white mycelium of the fungus [10]. FBR has a global distribution and can cause losses of up to 60%. It has been reported in many scientific papers and technical reports in Asia, Europe, America, and Africa [11]. Faced with this serious threat to onion yield and economic sustainability, stakeholders have identified solutions to manage the disease. These management solutions include chemical control, where specific compounds are used to fumigate or treat the soil prior to planting [12]. Other strategies include the use of resistant varieties and integrated management with the use of biological control agents [13]. Integrated management practices are essential to minimize the impact of FBR on crop production. Among them, using biological control agents such as arbuscular mycorrhizal fungi (AMF) and *Trichoderma* species make up one of the most promising alternative methods [14].

AMF has a mutualistic relationship with host plant roots [15], reducing plant need for fertilizers, promoting stress tolerance, and increasing its quality and yield [16]. AMFs are effective against root diseases and provide resistance against nematodes [17]. Among AMFs, *Funneliformis mosseae* (family Glomeraceae, formerly *Glomus mosseae* [18]) is the most ubiquitous and abundant in agricultural soils [19]. Several studies have reported that this strain can improve plant resistance to biotic stress against a wide range of phytopathogens, including *Fusarium* [20]. These beneficial effects are related to *F. mosseae’s* ability to promote plant development and growth while enhancing disease resistance (i.e., improving host plant barriers, disrupting pathogen structure, and shielding plant tissues) [21,22].

*Trichoderma* species have also been studied as alternatives to chemical control of several plant diseases with different mechanisms of action, such as antibiotic production and mycoparasitism, promotion of plant growth, and competition for nutrients and space [23]. These beneficial fungi provide a more sustainable and environmentally friendly approach to pest and disease management in agriculture. By reducing the reliance on chemical pesticides, they contribute to the promotion of integrated pest management (IPM) strategies, which aim to maintain a balance between pest control and environmental protection [24]. Among the *Trichoderma* genus, *Trichoderma harzianum* is one of the most frequently used species in the management of plant diseases [25].

Given the positive outcomes underlined for these biocontrol agents, we hypothesized that the combination of AMF with *T. harzianum* could be used as a suitable biocontrol agent against FBR disease. This study aimed to identify an effective FBR biocontrol agent. The fungi of the rotted onion bulbs were isolated, characterized, and tested for their pathogenicity in order to assess the causative agent of FBR. A greenhouse experiment was carried out to test the effectiveness of the beneficial fungi in controlling the disease. Both AMF (the *F. mosseae* pure strain and a commercial AM formulation) and *T. harzianum* treatments were used individually and in combination.

## 2. Results

### 2.1. Isolation and Characterization of Pathogenic Isolates

**Figure 1:**
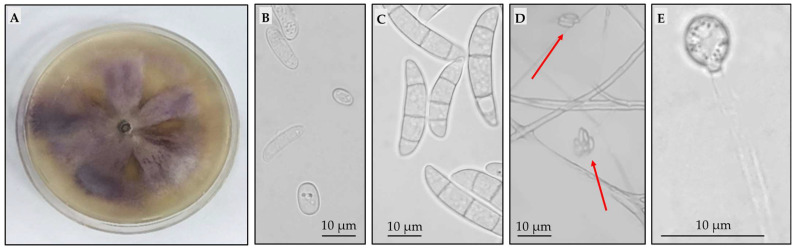
Macroscopic and microscopic characteristics observed for isolate S6, putatively identified as *Fusarium oxysporum*. (**A**) Violet pigmentation of aerial mycelium on potato dextrose agar. (**B**) Microconidia with non-segmented, oval, and kidney shapes. (**C**) Segmented macroconidia with three or four compartments. (**D**) False heads microconidia organization on monophialides. (**E**) Terminal chlamydospores.

The isolation of fungi from diseased plants resulted in five *Fusarium*-like isolates. Despite the use of the selective medium, other fungi were obtained from diseased plants. These isolates were putatively identified as *Mucor* and *Rhizopus*. Since these genera are not associated with FBR, they were not further processed in the investigation. Among the *Fusarium* isolates, four were purified and morphologically characterized. However, as they were not able to produce macro- and microconidia, they were not processed further. Isolate S6 was putatively identified as *F. oxysporum* based on the growth pattern and macroscopic and microscopic characteristics. Figure 1A shows the peculiar violet pigmentation of the aerial mycelium usually ascribed to the *Fusarium* genus. The other panels of Figure 1 report the microscopic characteristics associated with *F. oxysporum* species. Two types of asexual spores were observed, namely, microconidia with non-segmented oval or kidney shapes (Figure 1B) and segmented crescent (hook)-shaped macroconidia with three or four compartments (Figure 1C). Microconidia were also found to be organized on false heads on monophialides (Figure 1D), and the presence of a single terminal chlamydospore was observed (Figure 1E).

**Figure 2:**
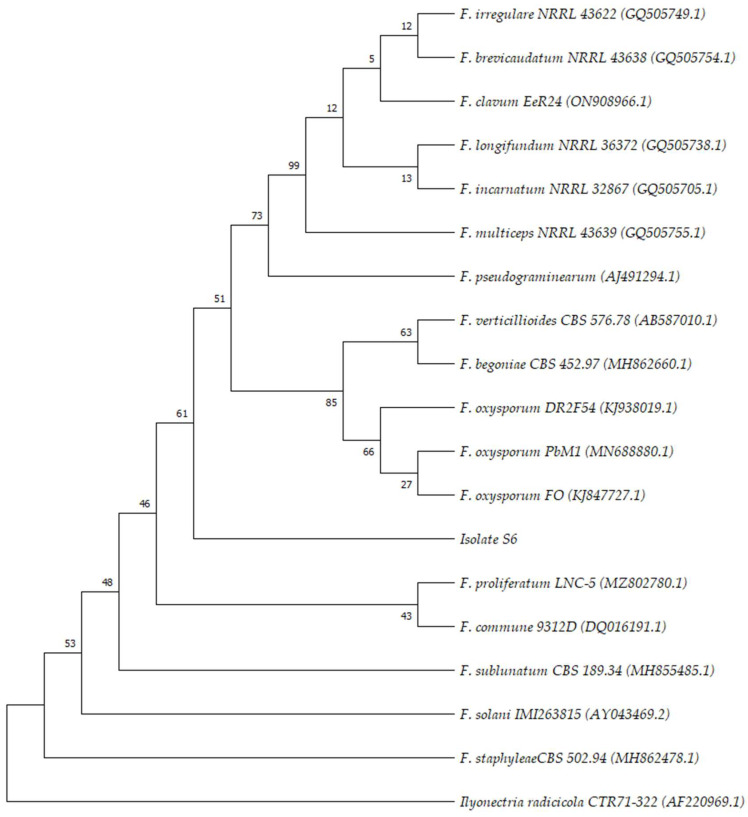
Bootstrap consensus phylogenetic tree inferred by using the Maximum Likelihood method and Kimura 2-parameter model. The evolutionary history of the species under study is assumed to be represented by the bootstrap consensus tree generated from 1000 repetitions [26]. Branches associated with partitions that were replicated in fewer than 50% of bootstrap replicates are collapsed. Below the branches, the proportion of duplicate trees where the connected taxa grouped together in the bootstrap test (1000 repetitions) is displayed [26]. By automatically applying the Neighbor-Join and BioNJ algorithms to a matrix of pairwise distances calculated using the Maximum Composite Likelihood (MCL) technique, and then choosing the topology with the best log likelihood value, the initial tree(s) for the heuristic search was created. Evolution was modeled with a discrete Gamma distribution.

ITS sequencing and phylogenetic analysis confirmed the identification of isolate S6 as a species belonging to the *F. oxysporum* complex. Figure 2 shows the phylogenetic tree inferred using several representative species of *Fusarium* and isolate sequence.

**Figure 3:**
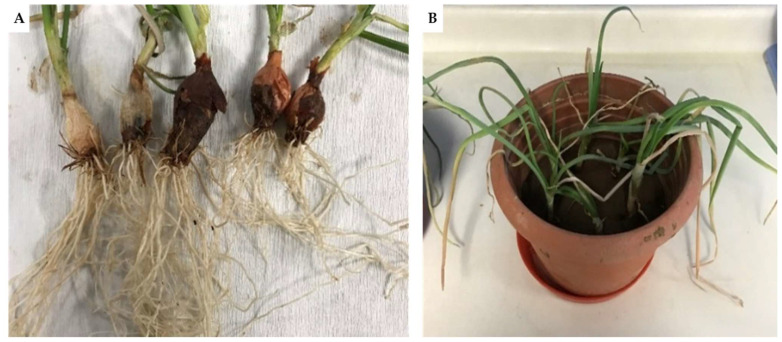
Onion bulbs (**A**) and aerial part (**B**) with evident symptoms of fusariosis.

After molecular identification, the isolate *F. oxysporum* S6 was investigated for its pathogenicity toward onion. Figure 3 shows the evident symptoms observed in infected plants. Within infected plants, the presence of isolate was confirmed by culturable approaches. Based on these results and the absence of pathogenesis on the other tested plants (i.e., *S. lycopersicum* and *Z. mays*), the isolate was identified as *F. oxysporum* f. sp. *cepae* S6.

### 2.2. Pathogenicity Tests of Susceptible Onion Variety

Table 1 reports the results obtained from onion varieties susceptibility test to FOC infection. Among the local varieties tested, Şampiyon was determined to be the most resistant, with a disease severity rate of 27%. Conversely, Gence was determined as the most susceptible variety, with a disease severity rate of 71%. The Seç variety recorded average values of disease severity (43%).

### 2.3. Greenhouse Disease Biocontrol Experiment

#### 2.3.1. Disease Evaluations

After four weeks, plants in the greenhouse started to show evident symptoms of fusariosis, with a different disease pattern based on treatment. Table 2 shows the results of disease evaluations.

In the presence of FOC alone (P), disease severity obtained the highest values (90%). The co-occurrence of beneficial fungi, singularly and in combination, promoted a decrease in severity (73% on average) and a suppression rate ranging from 14% to 24%. The treatment with *F. mosseae* (FP) induced the best FOC control, with a disease severity of 69% and a disease suppression rate of 34% (*p <* 0.05). The improvements recorded in the other treatments were not statistically significant compared to the control (*p* > 0.05).

#### 2.3.2. Analyses of Plant Material

Microscopic observations of samples obtained from treatments, including AMF application, showed that all fungal structures, including extra- and intra-radical mycelia, vesicles, and fungal spores, could be observed in all colonized roots. The fungal arbuscules could also be observed in the root samples, but since these structures are not persistent for a long time, they will disappear after colonization, and the spores and vesicles will be formed in the next steps of colonization. For this reason, arbuscules were not visible in some of the root samples, depending on the time of sampling for microscopic observation. The frequency of vesicles, as well as fungal spores, is higher than the other fungal structures such as arbuscules. Intercellular hyphae and vesicles were also identified in the root cortex region. Table 3 reports the results of the colonization density, mycorrhizal dependency, and spore density obtained from treatments, including AMF application (i.e., F, M, FP, MP, FTP, and MTP).

The AMF colonization rates varied from 8.91 to 24.09%. The highest colonization rates were recorded in the presence of *F. mosseae* (*p* < 0.05). In the absence of the pathogen, the application of *F. mosseae* recorded a colonization rate of 20%. In the presence of the pathogen, an average colonization rate of 24% was recorded when this AMF was applied alone (FP) or in combination with T22 (FTP). In the absence of the pathogen, the colonization rate of commercial AMF (ERS, M experimental condition) was similar to F. However, in the presence of the pathogen, the lowest colonization rates were obtained for this product, both in the presence and absence of T22 (*p* < 0.05).

Mycorrhizal dependency also varied with treatment. In the absence of the pathogen, the exclusive presence of *F. mosseae* (F) recorded higher percentages than ERS (M). However, for both *F. mosseae* and ERS, there were no statistical differences between the presence and absence of the pathogen, even when applied in combination with T22 (same statistical grouping for F, FP, and FTP and M, MP, and MTP, *p* > 0.05).

Spore densities differed significantly between *F. mosseae* and ERS. The best values were observed in the F and FTP treatments, while the lowest values were observed in the ERS treatments (*p* < 0.05). While no statistical significance was observed among the ERS treatments (M, MP, and MTP, *p* > 0.05), in the presence of the pathogen, the application of *F. mosseae* in combination with T22 improved this parameter compared to the application of F. mossae alone (FP vs. FTP, *p* < 0.05).

**Figure 4:**
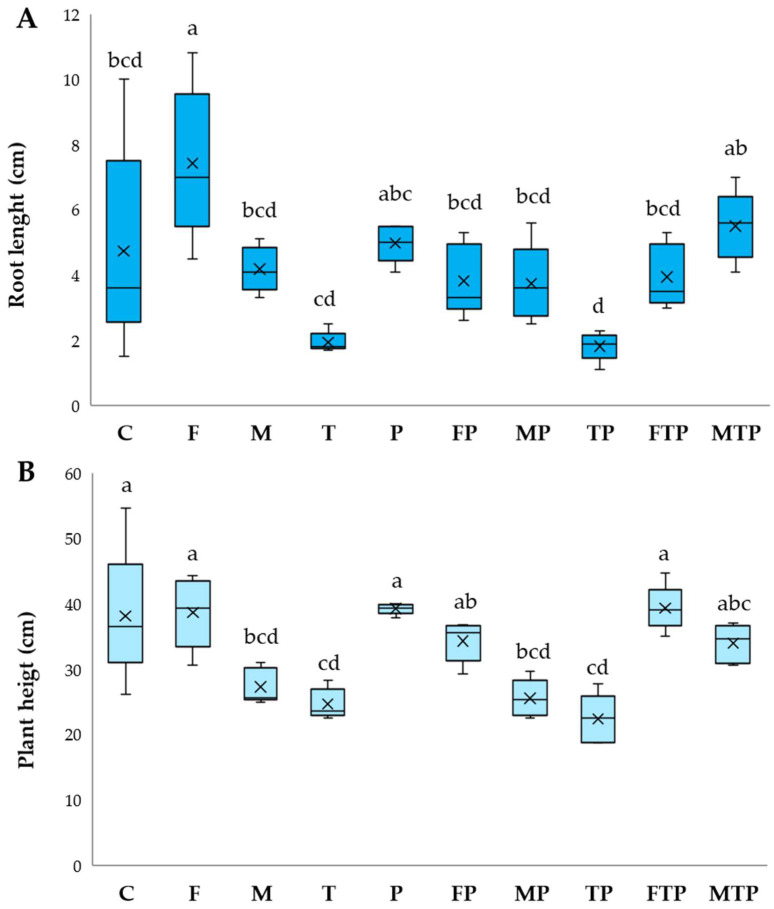
Root length (**A**) and plant height (**B**) registered under greenhouse disease control experiment. Results followed by different letters are significantly different according to Tukey’s post hoc test following ANOVA (*p* < 0.05). Control (C); *F. mosseae* (F); arbuscular mycorrhizal fungi − ERS (M); *T. harzianum* − T22 (T); *Fusarium oxysporum* f. sp. *cepae* − FOC (P); *F. mosseae* + FOC (FP); ERS + FOC (MP); T22 + FOC (TP); *F. mosseae* + T22 + FOC (FTP); ERS + FOC + T22 (MTP). The central horizontal line represents the median, the × symbol the mean, while whiskers indicate the range of values from the lowest to the maximum. The first and third quartiles are shown by the box’s upper and lower boundaries.

**Figure 5:**
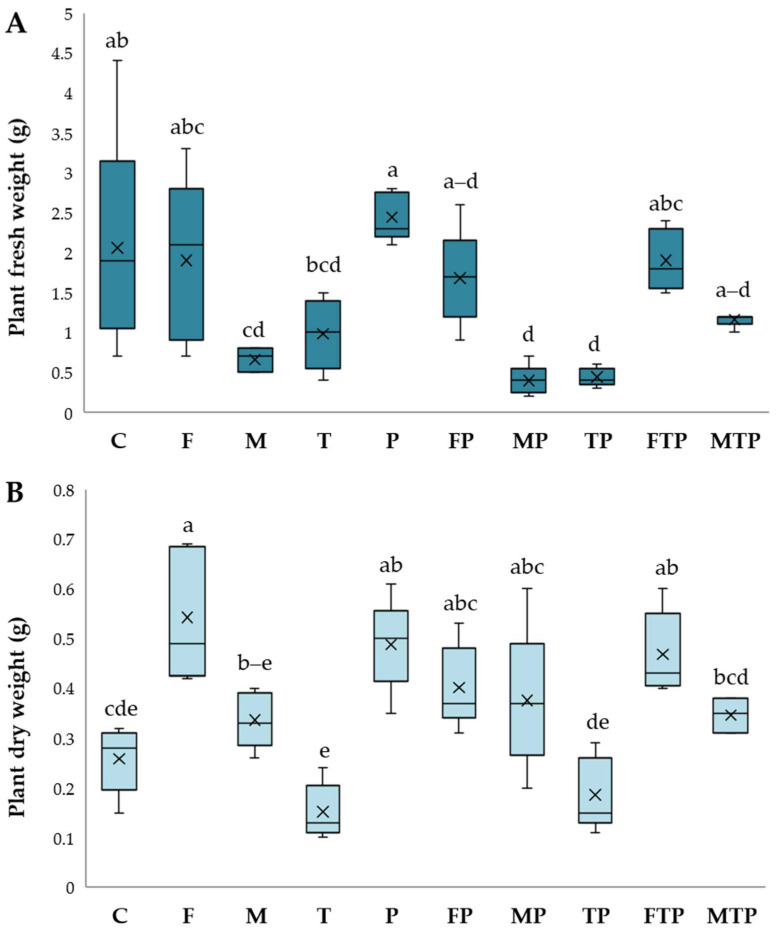
Plant fresh (**A**) and dry weight (**B**) recorded in plants grown under greenhouse disease control experiment. Results followed by different letters are significantly different according to Tukey post hoc test following ANOVA (*p* < 0.05). Control (C); *F. mosseae* (F); arbuscular mycorrhizal fungi − ERS (M); *T. harzianum* − T22 (T); *Fusarium oxysporum* f. sp. *cepae* − FOC (P); *F. mosseae* + FOC (FP); ERS + FOC (MP); T22 + FOC (TP); *F. mosseae* + T22 + FOC (FTP); ERS + FOC + T22 (MTP). The central horizontal line represents the median, the × symbol the mean, while whiskers indicate the range of values from the lowest to the maximum. The first and third quartiles are shown by the box’s upper and lower boundaries.

**Figure 6:**
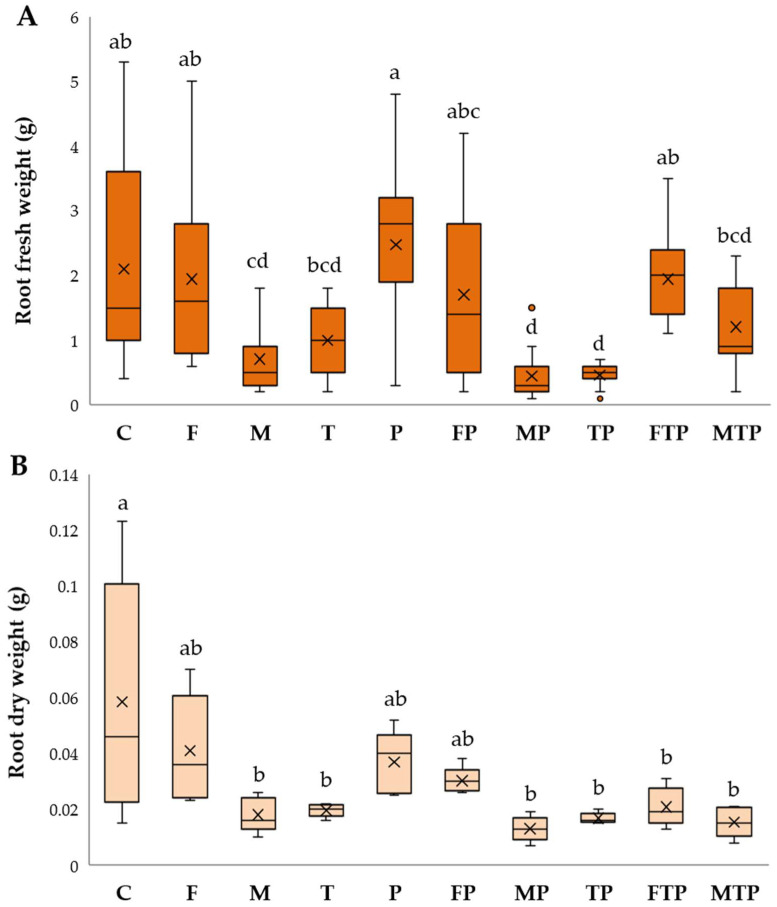
Root fresh (**A**) and dry weight (**B**) under greenhouse disease control experiment. Results followed by different letters are significantly different according to Tukey’s post hoc test following ANOVA (*p* < 0.05). Control (C); *F. mosseae* (F); arbuscular mycorrhizal fungi − ERS (M); *T. harzianum* − T22 (T); *Fusarium oxysporum* f. sp. *cepae* − FOC (P); *F. mosseae* + FOC (FP); ERS + FOC (MP); T22 + FOC (TP); *F. mosseae* + T22 + FOC (FTP); ERS + FOC + T22 (MTP). The central horizontal line represents the median, the × symbol the mean, while whiskers indicate the range of values from the lowest to the maximum. The first and third quartiles are shown by the box’s upper and lower boundaries.

Plant development was different based on treatment; results of growth parameters are given in Figure 4, Figure 5 and Figure 6. 

In the absence of pathogenesis, all growth parameters were positively influenced by *F. mossae’s* presence (F). For this treatment, the best plant height, dry weight, and root length were recorded (*p <* 0.05). Except for root dry weight, FOC disease spread in the presence of this beneficial strain (FP) was blocked, and *F. mossae* biocontrol promoted the development of plants similar to the control, especially in combination with T22 (FTP). Even with a lower efficacy than *F. mossae*, a good response was obtained with the commercial formulation, both in the pathogenesis absence (M) and presence (MP). As described for *F. mossae*, the combination with T22 (MTP) was successful. Applied alone, the T22 displayed no effects on plant development (T) or protection from FOC (TP).

Table 4 reports the phosphorus contents evaluated in onion plants.

Although the contents among the plants were not statistically different (*p >* 0.05), the treatment with AMF (FP and MP) showed improved accumulations of this element.

## 3. Discussion

In this study, the causal agent of onion basal rot was identified as FOC by common morphological, molecular, and pathogenesis testing approaches [27]. FOC is one of the most common *Fusarium* species that is usually found in this type of disease. Many authors have reported FOC as the causal agent of onion basal rot, with serious consequences for crops and yields [4,10]. The last update (May 2023) of the checklist of *Fusarium* species reported from Türkiye described a wide distribution of several fusariosis species in Türkiye [28], with consequent huge economic losses in the agricultural sector, which can record losses of up to 60% [11]. The presence of FOC in Türkiye has already been reported by other authors while investigating the *Fusarium* species associated with root and basal diseases of onions. According to a study by Bayraktar et al., the pathogen was detected in every field surveyed in the Ankara region and accounted for approximately 67% of all *Fusarium* isolates [29].

The use of resistant varieties is an effective tool being used by stakeholders to mitigate this pathogenesis [9]. However, new resistant varieties sometimes do not meet the requirements of farmers or consumers for the organoleptic qualities of the product [30]. This prompts research to test among susceptible varieties that may be less affected by pathogenesis in order to combine them with other sustainable tools and obtain yields appropriate to market demand. In our study, therefore, the results obtained for the three varieties grown in the study area may have a significant impact.

Among the other sustainable tools available, FOC biocontrol by beneficial fungi was tested in this study. The results obtained confirmed the ability of AMF to be viable agents against diseases induced by FOC. This inhibitory activity might be linked to several properties ascribed to AMF, including plant nutrition improvement, root morphology shaping, secondary metabolite release by plants enhancement, rhizosphere microenvironment improvement, direct competition with pathogens for space and nutrients, and plant resistance and defense mechanisms promotion [31]. As a result of the activation of defense mechanisms, the symbiotic association of AMF with plants increases the activity of hormones and enzymes, and a physical barrier that counteracts pathogens is created [32].

The biocontrol mechanisms of AMF shown by our results are consistent with previous reports by several authors and for different soil-borne pathogens. These biocontrol abilities have been reported since the last few years of the 20th century. Caron et al., in 1985, showed that *Fusarium oxysporum* f. sp. *radicis–lycopersici* in tomato was inhibited in the presence of *Glomus intraradices* under different growing media. Barragán et al., in 1996, reported that *Glomus* spp. were valid inhibition agents of onion *Sclerotium cepivorum*. In a more recent study, Akköprü et al. confirmed that Glomus spp. in tomatoes decreases the disease severity of *Fusarium oxysporum* f. sp. *lycopersici* by 48–92%. Many of these inhibition agents include the presence of *F. mosseae* (formerly known as *Glomus mosseae*). The inhibitory abilities of this species against *Fusarium* and a wide range of other fungal phytopathogens have been reported in several studies [20]. These beneficial effects are related to the ability of *F. mosseae* to promote plant development and growth while enhancing disease resistance (i.e., improving host plant barriers, disrupting pathogen structure, and shielding plant tissues) [21,22].

The application of AMF can be combined with other microorganism products to enhance pathogen inhibition. However, the combination of microorganisms cannot be random. According to how effectively they work, microbial-based inoculants can interact with each other in different ways. These interactions can be negative if the co-occurrence decreases the effects registered singularly, positive if the combined effect is equal to the sum of their independent effects, or synergistically if the independent positive effects are enhanced by the combination [33]. The combined application of *F. mossae* with *T. harzianum* for fusariosis control has been described by limited studies. Among them, the recent study by Ghanbarzadeh et al. reported an effective suppression of *Fusarium proliferatum*-induced onion basal rot. However, the same authors described that the presence of *T. harzianum* T100 negatively affected onion development due to AMF development inhibition [34]. The selection of the correct AMF-*Trichoderma* combination was also found to be crucial for fusariosis control in melon, with a reduction in the pathogen population of 1–1.5 log [35]. Due to the limited number of AMF-*T. harzianum* host–plant combinations that have been studied so far, studies on this topic have not yet clarified the basis for the variability of this combination [36].

The positive interactions found in our AMF-*T. harzianum* combination could be further studied to add knowledge to the field. Moreover, the T22 product could be used in future studies in combination with other commercial and experimental AMF to unveil its suitability as a biocontrol enhancer. Resistant varieties could be investigated for the presence of endophytes involved in their resistance. Additional research should focus on evaluating the effectiveness of the tested combinations on FOC under open field experiments and different pedoclimatic conditions. It is essential to consider environmental impacts when it comes to the success of biocontrol agents [37].

## 4. Materials and Methods

### 4.1. Plant and Beneficial Fungi

The onion varieties (Seç, Gence, and Şampiyon) were supplied by MNT Seed Company (Türkiye). The pure AMF *F. mosseae* strain was provided by culture collections at the Department of Plant Protection, Faculty of Agriculture, Van Yüzüncü Yıl University. The AMF commercial formulation (ERS, ERS Endo Roots, containing 78.85 propagules gr^1^ of *F. mosseae*. *Rhizophagus* spp., *Gigaspora margarita*, and *Glomus* spp., https://www.bioglobal.com.tr/product/endo-roots-soluble-ers, accessed on 18 September 2023) and *T. harzianum* commercial formulation (T22, T22-PLENTERBOX, containing 4 × 10^8^ spores g^−1^ of *T. harzianum* Rifai Irk KRL-AG2(T22), https://www.bioglobal.com.tr/product/t-22-planter-box, accessed on 18 September 2023) were supplied by Bioglobal (Antalya, Türkiye).

### 4.2. Isolation and Morphological Characterization of Pathogenic Isolates

Samplings were carried out in Polatlı, Ankara region, as the main onion production area during May–July 2019. The plants with disease symptoms were sampled randomly from different fields. A total of 35 diseased samples were collected. Isolation was performed on *Fusarium* selective medium [38] supplemented with streptomycin, neomycin, and 2,6-dichloro-4-nitroanaline, as previously described [39]. Briefly, small pieces of diseased tissue (~1 cm) were cut from the 35 samples. The pieces were treated with 0.5% sodium hypochlorite solution for 30 s, 70% ethanol solution for 20 s, and rinsed five times with sterile distilled water before drying. The dried pieces were placed on SFA and incubated at 25 °C for 5–10 days. Isolates were purified using single spore method [38], and morphological identification was performed using identification keys [40,41].

### 4.3. Pathogenic Isolates Molecular Characterization 

Isolate S6 was grown on PDA medium for 7–10 days for molecular characterization. A total of 250 mg of the mycelium was scraped and crushed with liquid nitrogen, and DNA extraction was performed using the Qiagen Plant Mini DNA Kit. Primers ITS-5 (5′GGA AGT AAA AGT CGT AAC AAG G 3′) and ITS-4 (5′-TCC TCC GCT TAT TGA TAT GC-3′) were used for amplification of ITS regions. The DNA amplifications were performed in a thermocycler (Techne TC-5000, Burlington, NJ, USA) using the following cycle parameters: initial denaturation at 96 °C for 5 min; 36 cycles of denaturation at 94 °C for 30 s, annealing at 52 °C for 30 s, and extension at 72 °C for 90 s; and a final extension step at 72 °C for 7 min (Cobos and Martin, 2008). Obtained PCR products were loaded onto 1.5% agarose gel and visualized by running electrophoresis in 1X TBE (40 mM Tris-borate, 1 mM EDTA, pH: 8.0) buffer. Sequence analyses were performed by BM (Gene Research and Biotechnology Company, Ankara, Türkiye). The nucleotide sequence results of the PCR products were analyzed using the BIOEDIT v. 7.2 software program.

The NCBI (National Center for Biotechnology Information) database was used to compare the sequence with those already in the database using the local base alignment search (BLAST). This was performed using the NCBI website (accessed on 15 September 2023) at http://www.ncbi.nlm.nih.gov. The similarity of the sequences considered was greater than 99%. The phylogenetic analysis was carried out using MEGA v. 11 software [42]. Bootstrap consensus phylogenetic tree was inferred using the Maximum Likelihood approach and Kimura 2-parameter model [43] following alignment and best model analysis.

### 4.4. Isolates Pathogenicity Tests and Susceptibility of Onion Varieties

To identify the forma specialis of FOC, a pathogenicity test was carried out on onion varieties (Seç, Gence, and Şampiyon), *Solanum lycopersicum,* and *Zea mays* (these species are hosts of many *F. oxysporum* formae speciales but are not infectable by FOC). A pure fungal culture was grown on PDA for 10 days at 25 °C. Spores were scraped from Petri dishes using 10–15 mL of distilled water and a sterile lancet. The spore suspension was then adjusted to 1 × 10^5^ spores mL^−1^ using a hemocytometer. Rooted seedlings of onions, tomatoes, and maize (first leaf developed) were cut (1–1.5 cm); dipped in the adjusted spore suspension; and left for 20 min. The control groups were treated according to the same procedure, except that sterile water was used for the soaking process instead of spore suspension. The infected and control seedlings were then transferred to 1 L pots containing a mixture of 1:1 peat and perlite. The pots were then transferred to a climatic chamber and incubated at 24 ± 2 °C, 70–80% humidity, and 12 h of light/dark conditions for 4 weeks. The experiments were arranged in a randomized block design with 5 replications per pot. After the disease appeared, isolation was repeated on diseased plants to confirm pathogenicity and assign forma specialis to FOC.

The susceptibility of onion varieties to the pathogen was investigated using the same experimental procedures after confirming the FOC forma specialis. After four weeks, the plants affected by the disease were sampled and investigated to confirm the presence of FOC and determine the disease severity using the following equation [44]:

Disease severity (%) = [Σ(S × L)/(M × Smax)] × 100

In the equation, S = scale value, L = the number of roots evaluated in the test, M = the total number of roots, and Smax = the highest scale value. The scale of 1–5 was used to determine the severity of disease caused by FOC in onion plants as follows [44]:

1. Healthy plant.
2. Brown on 1/4 of the roots.
3. Brown on 2/4 of the roots; slight rot.
4. Brown on 3/4 of the roots; moderate rot.
5. Completely browned or rotten roots.

### 4.5. Greenhouse Disease Biocontrol Experiment

Disease biocontrol trials were carried out at the Van Yüzüncü Yıl University, Faculty of Agriculture, Department of Plant Protection-Phytopathology in a greenhouse. Peat + perlite (1:1) mixture was prepared for the seedling-growing medium. Vermiculite was used as a cover cap. The pathogen inoculation trial was set up with ten different treatment groups according to a randomized plot design with five replications. The ten treatments considered were as follows:

1. Control (C);
2. *F. mosseae* (F);
3. ERS (M);
4. T22 (T);
5. FOC (P);
6. *F. mosseae* + FOC (FP);
7. ERS + FOC (MP);
8. T22 + FOC (TP);
9. *F. mosseae* + T22 + FOC (FTP);
10. ERS + FOC + T22 (MTP).

#### 4.5.1. AMF and Trichoderma Harzianum Inoculation

Pots were filled with mortar material consisting of a peat + perlite mixture (1:1 *w*/*w*). The different beneficial fungi were applied only once to the seedbed by mixing them with a mortar. The final density of *F. mosseae* was 250 spores g^−1^ soil. The application dose of commercial AMF was 2.5 g g^−1^ soil. The T22 application rate was 5 g per 50 onion plants (1 × 10^7^ spores/g) soil. 

Onion seeds were sowed on the seedbed, and the seeds were covered with a mixture of peat + perlite (1:1 *w*/*w*). Pots were incubated in a climatic chamber at 24 ± 2 °C, 70–80% humidity, and 12 h of light/dark conditions for 8 weeks. Onion seeds were irrigated with distilled water until germination and periodically after germination.

#### 4.5.2. Disease Induction

Four weeks after the application of *F. mosseae*, ERS, and T22, the rooted onion seedlings were removed from the pots, and infected and control groups were processed and incubated as described in Section 4.4 for 8 weeks.

#### 4.5.3. Disease Evaluations

After 4 weeks, the disease severity (%) was determined as described in Section 4.4. The disease suppression rate was also calculated as follows [45]:

disease suppression rate (%) = X × 100/Control disease severity

where X = Control disease severity − Treatment group disease severity

Isolation was repeated on diseased plants to confirm FOC presence.

#### 4.5.4. Analyses of Plant Material

Plants were harvested 8 weeks after inoculation (BBCH405, [46]) and investigated for their AMF colonization, spore density, mycorrhizal dependency, and several plant growth and biochemical parameters. 

The host plant root colonization by AMF was measured by root clearing and staining [47] and the Grid Line Intersect method [48]. The above-ground parts of onion plants were cu, and the root and root collar parts were separated from the soil, thoroughly washed under tap water, and the soil particles adhering to the roots were cleaned. Then, 1–0.5 g was taken from the roots and placed in acidified formal alcohol fixation liquid (90 mL of 70% alcohol, 5 mL of formaldehyde, and 5 mL of acetic acid) until dyeing. Dyed roots were stained with 0.05% Lactophenol blue (Merck KGaA, Darmstadt, Germany), and the roots were cleaned 2–3 times with lactoglycerol. Microscopic slides were prepared to observe fungal colonization and structures. 

Spore density was measured by isolating AMF spores from the soil samples (1 g in 3 replicates) using wet-sieving and centrifugation with the sucrose solution (55%) method [49] and 40× stereomicroscope (Leica, Wetzlar, Germany) counting. 

Mycorrhizal dependency, the extent to which a plant needs mycorrhizal conditions to grow to its maximum potential, was determined using the following equation [50]:

Mycorrhizal dependency (%) = [(A − B) A] × 100

Considering A is plant dry weight with AMF, and B is plant dry weight without AMF.

Several morphological and growth parameters, including dry and fresh weight (g), root dry and fresh weight (g), root length (cm), and plant length (cm), were measured. Root and green parts of the washed and cleaned plants were measured to obtain plant height and root length and weighed separately. Samples were dried at 70 °C, and after 48 h, the dry weights were estimated.

Dried samples were investigated for their total phosphorus content with a colorimetric method [51]. Briefly, 0.5 g of the extracts was weighed, and 1 mL of ethyl alcohol (Merck 818,760, Germany) was added and burned. Then, 4 mL of hydrochloric acid (Merck 1.05590.2500, Germany) was added to the samples and kept at 90 °C for 15 min. The extracts were filtered and measured by a spectrophotometer (Jenway 6505 UV/vis, Bibby Scientific Limited, Staffordshire, UK) at 430 nm.

### 4.6. Statistical Analysis

A one-way ANOVA was applied to test the effects of treatments on the investigated variables. Prior to ANOVA, data were screened by z-score visualization method, filtering out outliers in the presence of z-scores greater than +3 or less than −3, and tested for normality using Excel. In the presence of significant differences, separation of the means was performed by Tukey’s post hoc test at 5% level of significance (*p* < 0.05). Statistical analysis was performed by SPSS software version 26 (IBM Corp., Armonk, NY, USA).

## 5. Conclusions

In this work, we hypothesized that the combination of AMF and *T. harzianum* could be used as an effective biocontrol agent against FBR disease, given the excellent outcomes highlighted for both biocontrol agents. The pathogenic strain of FOC obtained from onions with evident basal rot disease was characterized and used in greenhouse biocontrol trials. The greenhouse biocontrol trials showed that the best pathogen suppression was obtained when the *F. mosseae* pure strain was used alone (a disease suppression rate of 23.55%). The *F. mosseae* pure strain also showed the best plant growth promotion. Good biocontrol was also achieved with the commercial AMF formulation of *F. mosseae* in combination with *T. harzianum* (a disease suppression rate of 21%). The positive interactions found in the AMF-*T. harzianum* combinations could be further studied to add knowledge to the field. In addition, the T22 product could be used in future studies in combination with other commercial and experimental AMF to unveil its suitability as a biocontrol enhancer. Resistant varieties could be investigated for the presence of endophytes involved in their resistance. Further research should focus on evaluating the efficacy of the tested combinations on FOC under open field experiments and different pedoclimatic conditions. The findings obtained suggest an interesting potential application in the management of FOC in onion crops. Considering the global distribution of FBR disease and the need for integrated management practices, our results are relevant. The results obtained add knowledge to the field and provide a valid basis for future research studies. The study of commercial products was useful to validate the results obtained with the experimental AMF. In addition, the results obtained with these commercial products are useful to be able to directly transfer the results to farms. This aspect is particularly relevant in terms of knowledge transfer and potential returns to applied research and stakeholder networking in the agricultural supply chain.

## Figures and Tables

**Table 1:** Disease severity of *Fusarium oxysporum* f. sp. *cepae* (FOC) in onion varieties.

Variety	Disease Severity (%)
Gence	70.7 ± 5.0 a
Seç	42.7 ± 1.6 b
Şampiyon	26.7 ± 2.1 c

Results followed by different letters are significantly different according to Tukey’s post hoc test following ANOVA (*p* < 0.05).

**Table 2:** Disease severity (mean ± standard deviation) and disease suppression rate in onion plants inoculated with FOC.

Treatment	Disease Severity (%)	Disease Suppression Rate (%)
P	90 ± 4.45 a	
FP	68.88 ± 5.73 b	23.55
MP	75.55 ± 4.17 ab	16.05
TP	77.77 ± 8.62 ab	13.58
FTP	70.55 ± 2.43 ab	21.61
MTP	71.10 ± 2.73 ab	21.00

Results followed by different letters are significantly different according to Tukey’s post hoc test following ANOVA (*p* < 0.05). *Fusarium oxysporum* f. sp. *cepae* − FOC (P); *F. mosseae* + FOC (FP); arbuscular mycorrhizal fungi − ERS + FOC (MP); *T. harzianum* − T22 + FOC (TP); *F. mosseae* + T22 + FOC (FTP); ERS + FOC + T22 (MTP).

**Table 3:** AMF colonization rates, mycorrhizal dependency, and AMF spore density (means ± standard deviations) recorded for plants subjected to mycorrhizal treatment, with and without pathogens.

Treatment	Colonization Rate (%)	Mycorrhizal Dependency (%)	Spore Density (Spores 10 g^−1^)
F	20.85 ± 2.150 ab	51.98 ± 4.25 a	50.40 ± 1.36 a
M	20.86 ± 1.66 ab	23.58 ± 5.58 bc	17.2 ± 1.07 c
FP	24.078 ± 0.96 a	36.02 ± 4.69 abc	34.2 ± 2.94 b
MP	14.60 ± 1.87 bc	18.33 ± 3.24 c	21.8 ± 1.68 c
FTP	24.09 ± 0.69 a	44.39 ± 6.69 ab	48.6 ± 2.32 a
MTP	8.91 ± 0.68 c	23.50 ± 4.39 bc	16.4 ± 1.50 c

Results followed by different letters are significantly different according to Tukey’s post hoc test following ANOVA (*p* < 0.05). *F. mosseae* (F); arbuscular mycorrhizal fungi − ERS (M); *F. mosseae* + *Fusarium oxysporum* f. sp. *cepae* − FOC (FP); ERS + FOC (MP); *F. mosseae* + T22 + FOC (FTP); ERS + FOC + T22 (MTP).

**Table 4:** Onion plant phosphorus element values (mean ± standard deviation) according to treatment groups.

Treatment	Phosporous Content (mg Kg^−1^)
C	0.78 ± 0.19
F	0.71 ± 0.06
M	0.88 ± 0.10
T	0.71 ± 0.06
P	0.61 ± 0.08
FP	1.01 ± 0.09
MP	0.94 ± 0.12
TP	0.81 ± 0.18
FTP	0.78 ± 0.06
MTP	0.74 ± 0.16
Tukey post hoc test	ns

Control (C); *F. mosseae* (F); arbuscular mycorrhizal fungi − ERS (M); *T. harzianum* − T22 (T); *Fusarium oxysporum* f. sp. *cepae* − FOC (P); *F. mosseae* + FOC (FP); ERS + FOC (MP); T22 + FOC (TP); *F. mosseae* + T22 + FOC (FTP); ERS + FOC + T22 (MTP); ns, not significant (*p >* 0.05).

## Data Availability

The datasets generated and/or analyzed during the current study are available from the corresponding authors on request.
